# Multicenter randomized trial of cell therapy in cardiopathies – MiHeart Study

**DOI:** 10.1186/1745-6215-8-2

**Published:** 2007-01-18

**Authors:** Bernardo R Tura, Helena F Martino, Luis H Gowdak, Ricardo Ribeiro dos Santos, Hans F Dohmann, José E Krieger, Gilson Feitosa, Fábio Vilas-Boas, Sérgio A Oliveira, Suzana A Silva, Augusto Z Bozza, Radovan Borojevic, Antonio C Campos de Carvalho

**Affiliations:** 1 Instituto Nacional de Cardiologia Laranjeiras, Rio de Janeiro, Brazil; 2 Instituto do Coração da Universidade de São Paulo, São Paulo, Brazil; 3 Centro de Pesquisa Gonçalo Muniz-Fiocruz, Salvador, Brazil; 4 Hospital Pró-Cardíaco, Rio de Janeiro, Brazil; 5 Hospital Santa Izabel-Santa Casa de Misericórida da Bahia, Salvador, Brazil; 6 Universidade Federal do Rio de Janeiro, Rio de Janeiro, Brazil

## Abstract

**Background:**

Cardiovascular diseases are the major cause of death in the world. Current treatments have not been able to reverse this scenario, creating the need for the development of new therapies. Cell therapies have emerged as an alternative for cardiac diseases of distinct causes in experimental animal studies and more recently in clinical trials.

**Method/Design:**

We have designed clinical trials to test for the efficacy of autologous bone marrow derived mononuclear cell therapies in four different cardiopathies: acute and chronic ischemic heart disease, and Chagasic and dilated cardiomyopathy. All trials are multicenter, randomized, double-blind and placebo controlled. In each trial 300 patients will be enrolled and receive optimized therapy for their specific condition. Additionally, half of the patients will receive the autologous bone marrow cells while the other half will receive placebo (saline with 5% autologous serum). For each trial there are specific inclusion and exclusion criteria and the method for cell delivery is intramyocardial for the chronic ischemic heart disease and intracoronary for all others. Primary endpoint for all studies will be the difference in ejection fraction (determined by Simpson's rule) six and twelve months after intervention in relation to the basal ejection fraction. The main hypothesis of this study is that the patients who receive the autologous bone-marrow stem cell implant will have after a 6 month follow-up a mean increase of 5% in absolute left ventricular ejection fraction in comparison with the control group.

**Discussion:**

Many phase I clinical trials using cell therapy for cardiac diseases have already been performed. The few randomized studies have yielded conflicting results, rendering necessary larger well controlled trials to test for efficacy of cell therapies in cardiopathies.

The trials registration numbers at the NIH registry are the following: Chagasic cardiomyopathy (NCT00349271), dilated cardiomyopathy (NCT00333827), acute myocardial infarction (NCT00350766) and Chronic Ischemic Heart Disease (NCT00362388).

## Background

Mortality from acute myocardial infarction has decreased dramatically over the years due to improved interventional therapy and adjunctive pharmacological treatment. On the other hand the incidence of heart failure has grown steadily reaching epidemic proportions. At its advanced stages, heart failure leads to a poor quality of life with severe restrictions to patients' daily activities and high costs to the health system in face of the constant need for hospitalizations.

The pathological basis of heart failure is cardiomyocyte death, which may compromise the efficiency of the heart as a pump. End-stage heart failure usually requires more aggressive treatment such as heart transplantation, a rather limited therapeutic option because of donor shortage and high medical costs. In this scenario, new therapies are clearly needed and cell-based therapies emerge as an alternative.

There is growing evidence from animal experimental studies and initial clinical trials that cell therapy may be effective in ameliorating the burden of different cardiopathies – for a review see [1]. In this article, we describe the design of an ongoing clinical trial sponsored by the Brazilian Ministry of Health, aiming to test the efficacy of bone marrow-derived cell therapies in the treatment of four different cardiopathies.

The Multicenter Randomized Cell Therapy Trial in Cardiopathies (MiHeart) is, in fact, composed of four independent clinical trials each one dealing with a specific cardiopathy, namely: Chagasic cardiomyopathy (NCT00349271), dilated cardiomyopathy (NCT00333827), acute myocardial infarction (NCT00350766) and chronic ischemic heart disease (NCT00362388).

Each clinical trial is coordinated by a so-called "anchor center", a medical institution with recognized undisputable tradition in clinical research, and a variable number of collaborating centers that have to be licensed by the anchor center. The collaborating centers are required to have large experience in the treatment of the targeted disease and will participate in selection and follow-up of the patients enrolled, as well as, after adequate training, in performing the autologous bone-marrow stem cell implant. A center may participate in more than one clinical trial as long as it meets the requirements set by the anchor centers. However, a given institution can only anchor one of the clinical trials. Overall there are 40 Institutions participating in the study. The study has a National Coordination, responsible for the online data acquisition and analysis of all four clinical trials through electronic case report forms (CRFs), located at the National Cardiology Institute, an institution directly affiliated to the Ministry of Health. The Steering Committee of the study is composed by two members of each anchor center, two members of the central coordination, two representatives of the Ministry of Health and two representatives of the Ministry of Science and Technology. There is also a Safety Committee composed of six medical doctors, of which at least three are cardiologists, and who are not involved in any aspect of the clinical trials.

The main hypothesis of this study is that patients who receive the autologous bone-marrow stem cell implants will have a mean absolute increase of 5% in left ventricular ejection fraction compared with the control group, 6 months after the procedure.

## Methods/Design

All 4 trials were designed as multicenter, randomized, double-blind, and placebo-controlled. The primary endpoint, common to all four trials, is the difference between LV ejection fraction (determined by Simpson's rule) at six and twelve months after intervention and baseline. Secondary endpoints are:

• Death by any cause within 1 year of intervention.

• Maximum oxygen consumption difference, as measured by ergoespirometry, at six and twelve months compared to baseline.

• Difference in quality of life as assessed by the Minnesota Living with Heart Failure Questionnaire at six and twelve months and baseline.

• Difference in NYHA functional class at six and twelve months and baseline.

• Percentage of patients that reached an absolute increase of 5% in LV ejection fraction at six and twelve months.

• Cost-effectiveness of autologous bone-marrow stem cell implant in comparison with the control group in one year.

For all clinical trials the safety endpoints are:

• Incidence of arrhythmias and conduction disturbances:

○ Number of ventricular extra systoles in six months and one year.

○ Number of sustained ventricular tachycardia episodes in six months and one year.

○ Number of non sustained ventricular tachycardia episodes in six months and one year.

○ Incidence of atrial fibrillation in six months and one year.

○ New-onset of atrioventricular or intraventricular conduction disturbances.

○ Need for artificial pacemaker implantation.

Each clinical trial has also tertiary endpoints which are not described in this article but are currently available at the NIH site.

The major inclusion criteria in all 4 studies are summarized in Table 1. Other inclusion and exclusion criteria for each specific study are not displayed in the Table but can be assessed at the NIH site.

**Table 1:** Major inclusion criteria for each of the four cardiopathies.

Inclusion Criteria	Dilated Cardiomyopathy	Chagas Disease	Chronic Ischemic Heart Disease	Myocardial Infarction
Age (years)	18 to 65	18 to 75	30 to 75	30 to 80
Diagnosis of target disease	WHO criteria	2 serologic test	typical angina with positive cardiac scintigraphy or MRI	WHO criteria
Congestive heart failure	III – IV NYHA	III – IV NYHA	no	no
Ischemia	no	no	II to IV CCS	not mandatory
Left ventricular ejection fraction	≤35%	≤35%	25%–55%	<50%
Method	Simpson	Simpson	Simpson	Dodge
Medical Therapy	Optimized	Optimized	Optimized	Optimized

The sample size calculation for the trials was based on the following assumptions:

• Based on data analysis of patients treated in the National Cardiology Institute with heart failure and in NYHA class III and IV in the last two years, we estimated a Gaussian distribution for the left ventricular ejection fraction with a mean value of 25% and standard deviation of 10%. These values were taken as reference for the dilated and Chagasic patients.

• Based on data analysis of patients treated at the Procardíaco Hospital with ischemic heart disease and class II-IV CCS in the last two years, we estimated a Gaussian distribution for the left ventricular ejection fraction with a mean value of 38% and a standard deviation of 14.1%. These values were taken as reference for the ischemic patients.

• After examining the literature it is reasonable to assume an improvement of 5% in ejection fraction after cell therapy.

• A panel of specialists evaluated that for the type of patient enrolled in this study a gain of 5% in ejection fraction could result in a significant clinical improvement.

Based on these assumptions, we estimate that to detect a 5% difference in ejection fraction with 95% confidence (1-α) and 80% power (1-β) and a standard deviation of 15% (a larger sd was used to accommodate for the multicenter character of the trial), we will need 142 patients. Considering a 5% loss within 12 months of follow-up, we ended up with 150 patients in each study arm and 300 patients in each trial.

All patient data are filed online by e-CRFs through software specially developed for each trial according to Title 21 Code of Federal Regulations – part 11 [2]. The data storage uses four mirror servers, two as web-servers and two as database servers. The whole computational system is duplicated in another area of the city. Detailed protocols, all the CRFs, the informed consent and standard operational procedures for marrow aspiration, mononuclear cell processing and catheterization for cell delivery are available in Portuguese [3].

Software was created in R version 1.9.0 to specifically generate the randomization sequence for the study. It is known that a simple randomization is sufficient and efficient to render the study groups homogeneous for known and unknown factors in large scale clinical trials. However in smaller trials restriction techniques are recommended. To insure homogeneity in the two arms of the study we adopted block randomization. We will use randomization by variable size block (blocks of 2, 4 or 6 patients). Patients are randomized after bone marrow aspiration and only the hematologist responsible for cell separation has the login and password to randomize the patient online. According to the assigned group, darkened syringes containing the mononuclear cell fraction or saline with 5% autologous serum are then prepared and sent for implant into the patients.

## Discussion

Many phase I clinical trials using cell therapy for cardiac diseases have already been performed. In Chagasic cardiomyopathy, we recently published the results of such a trial involving 28 patients in Brazil [4]. In dilated cardiomyopathy we published a case report [5] and just finished inclusion of 30 patients. This study showed the procedure to be feasible and safe, leading us to proceed with the larger randomized trial. In chronic ischemic heart disease we have published four reports using different injection routes and demonstrating the procedure to be safe and potentially efficacious [6-9]. The largest number of phase I clinical trials have been published in the setting of acute myocardial infarction. A number of randomized studies have also been performed in AMI [10-12] yielding conflicting results, and thus justifying the need for larger well controlled trials to test for efficacy of cell therapies in all cardiopathies.

## Competing interests

The author(s) declare that they have no competing interests.

## Authors' contributions

BRT and ACCC designed the general study, performed sample calculations and wrote the manuscript. HFM and AZB designed the dilated cardiomyopahty protocol. LHG, JEK and SAO designed the chronic ischemic disease protocol. RRS, GF and FVB designed the chagasic cardiomyopathy protocol. HFD, SAS and RB designed the acute myocardial infarction protocol.
